# Using large language models to extract information from pediatric clinical reports

**DOI:** 10.1371/journal.pdig.0000919

**Published:** 2025-07-23

**Authors:** Katharina Danhauser, Yingding Wang, Christoph Klein, Uta Tacke, Larissa Mantoan, Laura Aurica Ritter, Florian Heinen, Chiara Nobile, Moritz Tacke

**Affiliations:** 1 Department of Pediatrics, LMU University Hospital, Munich, Germany; 2 University Children’s Hospital, Basel, Switzerland; Mayo Clinic Arizona, UNITED STATES OF AMERICA

## Abstract

Most medical documentation, including clinical reports, exists in unstructured formats, which hinder efficient data analysis and integration into decision-making systems for patient care and research. Both fields could profit significantly from a reliable automatic analysis of these documents. Current methods for data extraction from these documents are labor-intensive and inflexible. Large Language Models (LLMs) offer a promising alternative for transforming unstructured medical documents into structured data in a flexible manner. This study assesses the performance of large language models (LLMs) in extracting structured data from pediatric clinical reports. Nine different LLMs were assessed. The results demonstrate that both commercial and open-source LLMs can achieve high accuracy in identifying patient-specific information, with top-performing models achieving over 90% accuracy in key tasks.

## Author summary

Big parts of today’s medical documentation is in the form of unstructured documents like medical reports. It is currently difficult to process these documents automatically, as, up to now, computers had problems to extract information from human-readable, unstructured text. This presents a problem for patient management and for medical research. A way to process medical documents in a reliable, automated way might therefore be helpful. In the last few years, several large language models were developed. These models are computer programs which can understand texts that are written in languages like English or German. In the current study, we tried to find out whether these large language models can extract information from medical reports. We used fictional but realistic documents about pediatric patients and tested several large language models. The results were promising, the best models performed well in most of the tasks assigned.

## Introduction

The majority of medical documentation is written for human readers in a form of unstructured text documents like clinical reports. Therefore, a wealth of clinical information is currently documented in a way that is not well suited for an automatic, computer-based analysis. This limits interoperability, delays data retrieval, and complicates the integration of patient information into electronic health records or research databases. A reliable way to process medical documents like clinical reports has the potential to improve both patient care and medical research. Specialized text processing systems [1] have proven valuable to scan archived text documents. However, their use is limited by a lack of flexibility. For a given task, the implementation of a successful text mining application includes several manual steps, e.g. the definition of rules which can be applied to extract items for the text, or the labeling of data which is necessary to train classical machine learning algorithms.

In contrast to these specific systems, large language models (LLMs) are a non-specific artificial intelligence (AI) tool for text processing [2]. The predecessors of the current LLMs exist since about 2018. In November 2022, the AI company OpenAI published ChatGPT, a LLM-based chatbot, drawing public attention. Unlike traditional methods, LLMs excel in understanding and contextualizing natural language, enabling them to process heterogeneous texts flexibly and accurately.

LLMs are trained on huge datasets including medical data. They therefore encode medical knowledge that can be used for data extraction tasks which require some amount of background knowledge, e.g. the ability to identify a viral pneumonia as an infectious disease. However, the use of LLMs in the medical domain carries some risks [3]. LLMs have no guarantee of a correct output. So-called “hallucinations”, i.e. LLM-generated text that contains wrong information, are a typical behavior of LLMs [4]. This might lead to incorrect decisions [5]. Furthermore, many LLMs are hosted by companies that are not part of the health system. The data protection level required for medical purposes might not be given in these cases, and the use of these services from within the health system not be compliant to the local laws. On-premise LLMs that are hosted on hospital infrastructure are an option to meet data protection requirements.

LLMs utilize an input text, known as the prompt, to generate an output text. To do so, they analyze the prompt, and it is justified to say that they “understand” it. Users can provide more information in natural language to instruct the output text to be formatted in a “classical” machine-readable format (that can be processed by a computer program without text processing capabilities). This allows them to create structured data from unstructured text, enabling the processing of medical documents like clinical reports.

General, i.e. not task-specific LLMs can process their input text without prior training (“zero-shot” learning). Performance can be enhanced by the addition of some examples to the prompt (“few-shot” learning) [6]. This approach may provide a faster and more flexible way to extract structured information from unstructured medical documents. First results have been promising [7]. The approach has e.g. be used to extract patient characteristics from published case reports [8].

This study evaluated how well LLMs can transform pediatric clinical reports into structured, machine-readable data. Both locally run LLMs and commercially available LLMs that can be accessed online were analyzed. The performance of the LLM on relevant tasks which include plain data extraction, simple calculations, classifications which requires some medical background knowledge, and the combination of several classifications was examined.

## Materials and methods

### Data set

A total of 100 fictitious clinical reports was used. They were inspired by the cases that were admitted as inpatients or outpatients Munich and Basel University Children’s Hospital. The fictional clinical reports were designed to mimic real-world variability, including diverse writing styles, medical terminologies, and complexity levels (e.g., ranging from single-page summaries to multi-page detailed reports).

The letters were given as plain text and did not include any formatting or letterheads. The letters were written in German. To respect data privacy, only fictional doctor’s letters were used for this study.

### LLMs

Several LLMs were evaluated. These LLMs were chosen to evaluate the influence of several factors for the given task, namely the model size, if known (e.g. 70B for a model with seventy billion parameters) as well as domain-specific and language-specific fine-tunes of the LLM. These included two commercially available remote LLMs (GPT-4o, version gpt-4o-2024-08-06, and GPT-4o-mini, version gpt-4o-mini-2024-07-18, both accessed via OpenAI), and several LLMs that can be run on local hardware:

- Base models:
  - Llama-3.1, with 8B and 70B parameters
  - Llama-3.2, with 1B parameters
  - Qwen 2.5, with 32B parameters
- Models that were fine-tuned for the medical domain
  - Llama3-Med42, with 70B parameters [9]
  - Meditron3, with 8B parameters
- A model which is fine-tuned for the German language:
  - Sauerkraut-3.1-8B

All local LLMs were run with a Q6 quantization in GGUF format. Up to two NVIDIA A100 GPUs with 40 GB VRAM were used for inference. All local models were accessed using the llama-cpp-python library version 0.3.1. OpenAI models were queried using the OpenAI API. The output of the LLMs was constrained to follow a JSON schema using the respective functionalities for the given models. The LLM temperature was always set to zero.

### Question categories

The LLMs were prompted to evaluate the letters in several categories: age (in months and in years), body weight, involved sub-specialties, symptoms upon admission, presence of infectious or genetic diseases, and eligibility for a hypothetical study involving children with infectious diseases. Specific instructions for every category were provided in the LLM’s prompt. All categories and their instructions are shown in Table 1.

**Table 1:** Prompts for the different question categories. Every prompt ended with the phrase The JSON variable should have the name XXX where XXX was a suited word (e.g. bodyweight_kg)

Category	Prompt
Body weight	The body weight in kilogram should be returned.
Age	The age at admission in completed months (or completed years) should be returned.
Main Sub-specialties	The main sub-specialties should be returned as a list. Valid values are, followed by a list of all pediatric sub-specialties
Symptoms at admission	The symptoms on admission should be returned as a list. Valid values are, followed by a list of all symptoms that occurred in any doctor’s letter
Genetic disorder	It should be assessed whether the patient has a probable or proven genetic condition, or not. Valid values are: True (if proven or probable) or False.
Infectious disease	It should be assessed whether an infectious disease is proven or probable as the reason of the admission. Valid values are: True (if proven or probable) or False.
Patient for study	It should be checked if the patient is a candidate for a study. Inclusion criteria are: - Age between 2 and 5 years (inclusive) - Admission due to proven or probable infection. Possible values: True (if candidate) or False.

The evaluation of the category Patient for study was conducted in two different ways. The LLM was directly prompted to directly classify whether a given patient could be included in the study (Patient for study/direct). The two inclusion criteria (age and admission due to an infectious disease) are separate categories. The additional category Patient for study/calculated classified a patient as a possible candidate if the results for Age and Infectious disease matched the inclusion criteria.

### Prompting

Few-shot prompting was used. For every letter, a set of four different example letters was randomly selected. The example letters were added to the prompt, along with the correct results for all categories (in JSON format). This prompt was used to analyze all the other letters. Therefore, each letter in the data set was analyzed nine times, with a different set of example letters each time.

The final prompt for querying a letter consisted of:

**Table:** 

Item	Contents
Preamble	This program extracts some information from doctors letters.
Category sub-prompts	The sub-prompts for all categories
Example preamble	Here are some examples:
Examples	Every letter from the example set, along with its correct results as a JSON object
Finishing clause	Here comes the doctor’s letter:
Query letter	The text of the query letter

Chain-of-thought prompting [10] was applied for those categories where a structured reasoning approach was seen as an option to improve performance. These categories were both age categories, the main sub-specialties, as well as the inclusion into the study.

In chain-of-thought calls, a different JSON schema was used. In the plain calls, the JSON objects contained only the results for the different categories (e.g. ’age_years’ : 0). In a chain-of-thought-call, the JSON object contained the reasoning steps and the result, e.g:

{’steps’:

[{’explanation’:

’The birthday is 2020-02-10 and the presentation day is

2020-02-14, therefore the age is 0 completed years.’,

’output’: {’age_years’: 0}}],

’final_answer’: {’age_years’: 0}}

These changed JSON structures were embedded in the example prompts. The LLM output was constrained to follow the same output format.

### Data processing

Model performance was measured for each category. If the LLM returned an invalid result (e.g. nothing or a string that was not machine-readable, i.e. not in a valid JSON format), all categories were considered incorrect. Otherwise, the results for a single letter were analyzed as follows:

- Body weight: Only errors of less than a gram were accepted
- Age in months/years: Only correct results were accepted
- Sub-specialties and Symptoms: Results were given in form of lists. The F1 score [11] was used to compare the true classifications and the results from the LLM
- Infectious disease, genetic disorder, study inclusion criteria: Only correct results were accepted

### Statistical evaluation

The aggregated results for the categories were analyzed as follows:

- Body weight, age values: Average number of correct results
- Sub-specialties and Symptoms: Average F1 value
- Infectious disease, genetic disorder, study inclusion criteria: The correct classifications were compared with the LLM results using the F1 score

Each category was normalized to a value between 0 and 100%. The total score for a given LLM was calculated as the average of all sub-scores. Bootstrap sampling [12] with 500 iterations was applied to estimate 95% confidence intervals for each metric, ensuring robust results despite the limited dataset size. The resampling process randomly drew from the dataset with replacement to create synthetic distributions.

## Results

**Fig 1:**
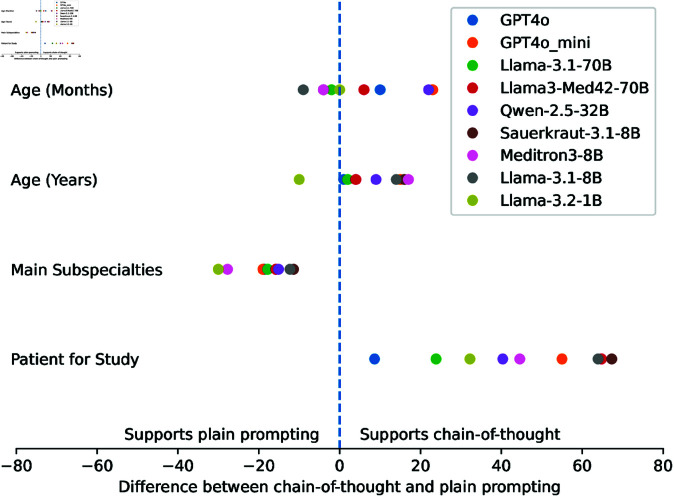
Comparison of chain-of-thought reasoning and plain prompting results. The x-axis represents the difference in scores achieved using chain-of-thought reasoning compared to plain prompting for each task.

In order to identify the categories which should be used to rank the models, a comparison between chain-of-thought-reasoning and plain reasoning was done for those categories where chain-of-thought-reasoning was seen as promising. The results are shown in Fig 1. Based on this result, chain-of-thought-reasoning was used for the age categories as well as for the study inclusion category in the following evaluations.

**Fig 2:**
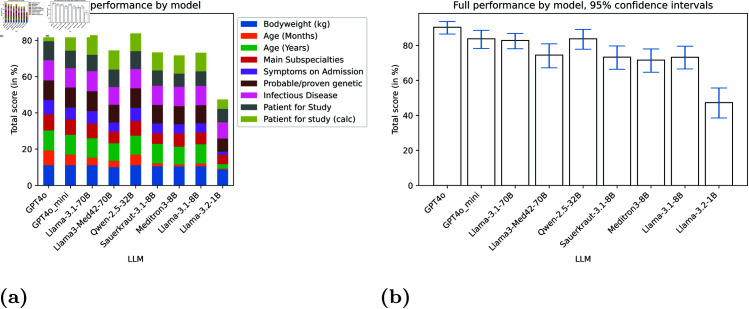
LLM comparison. Overall results for the different LLMs. Part (a) shows the contribution of the different question categories for all LLMs. Part (b) shows the 95% confidence intervals on the overall performance.

Fig 2a shows the total score for all models, including the contribution of each category to the final score. The 95% confidence intervals are depicted in Fig 2b as well as in Table 2. The maximum total score was achieved by GPT4o (90.40). Two of the larger local models (Llama-3.1-70B, Qwen-2.5-32B) along with GPT4o_mini 3.5 achieved scores above 80, all the others, except the smallest model Llama-3.2-1B had scores between 70 and 80.

**Table 2:** Overall performance of the different LLMs with 95% confidence intervals (CI).

Model	Score (Mean, CI)	
GPT4o	90.40	[86.55, 93.68]
GPT4o_mini	83.82	[78.26, 88.67]
Llama-3.1-70B	82.84	[78.14, 86.91]
Llama3-Med42-70B	74.50	[67.21, 80.92]
Qwen-2.5-32B	83.80	[77.86, 89.19]
Sauerkraut-3.1-8B	73.30	[66.38, 79.72]
Meditron3-8B	71.62	[64.68, 78.01]
Llama-3.1-8B	73.26	[66.58, 79.57]
Llama-3.2-1B	47.33	[38.53, 55.68]

**Fig 3:**
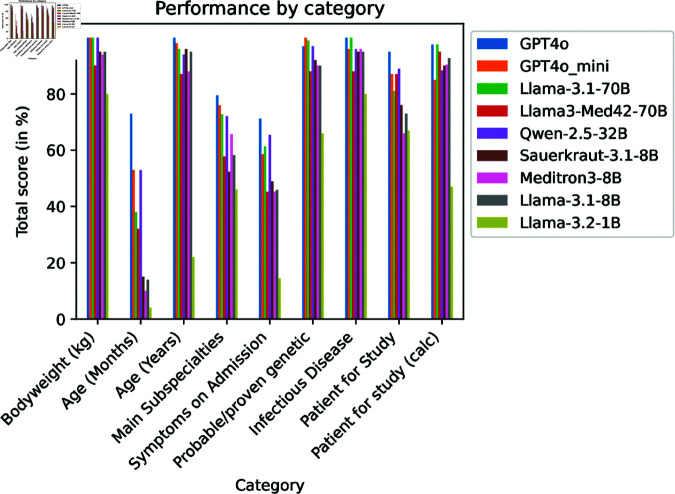
Question category comparison. Comparison of the LLM performance on the different question categories

The performance on the different categories is demonstrated in Fig 3. The highest scores for each task are shown in Table 3 and detailed in Table 4.

**Table 3:** Highest scores for the given categories.

Question category	Models	Score
Body weight	GPT4o/mini, Llama-3.1-70B, Qwen-2.5	100.00
Age (Months)	GPT4o	72.53
Age (Years)	GPT4o	100.00
Main Subspecialties	GPT4o	79.72
Symptoms on Admission	GPT4o	71.24
Probable / proven genetic	GPT4o_mini	100.00
Infectious disease	GPT4o, Llama-3.1-70B	100.00
Patient for study	GPT4o	95.12
Patient for study (calc)	GPT4o	97.59

**Table 4:** Mean performance of the models on the different question categories. Values printed in bold: highest values for the given category

Model	GPT4o	GPT4o_mini	Llama-3.1-70B	Llama3-Med42-70B	Qwen-2.5-32B
Body weight (kg)	**100.00**	**100.00**	**100.00**	89.81	**100.00**
Age (Months)	**72.53**	53.07	37.88	31.85	53.15
Age (Years)	**100.00**	97.93	95.93	87.12	94.12
Main Subspecialties	**79.72**	75.94	72.90	57.69	72.02
Symptoms on Admission	**71.24**	58.56	60.98	44.90	65.42
Probable/proven genetic	97.00	**100.00**	99.06	87.82	96.95
Infectious Disease	**100.00**	96.08	**100.00**	87.82	95.96
Patient for Study	**95.12**	87.16	81.10	87.17	89.02
Patient for study (calc)	**97.59**	85.17	97.49	94.98	87.94

Model	Sauerkraut-3.1-8B	Meditron3-8B	Llama-3.1-8B	Llama-3.2-1B	
Body weight (kg)	94.95	94.13	95.02	79.88	
Age (Months)	14.86	9.78	14.06	4.08	
Age (Years)	96.01	87.96	95.02	21.94	
Main Subspecialties	52.35	65.59	58.46	46.10	
Symptoms on Admission	49.40	45.16	45.82	14.36	
Probable/proven genetic	91.89	90.07	90.05	65.65	
Infectious Disease	95.04	95.98	94.92	79.81	
Patient for Study	76.03	65.82	72.75	66.70	
Patient for study (calc)	89.71	89.80	92.77	46.21	

The “body weight” task required a simple information extraction – the body weight was explicitly mentioned in the text. Here, all models performed well, and some perfectly well. The tasks that involved yes/no questions which required some medical knowledge (i.e. that a viral pneumonia is an infectious disease, and that the spinal muscular atrophy is a genetic disorder) produced similarly high scores, as did the age in years. The lowest performance was observed when the answer consisted of lists of classifications (the sub-specialties that are involved in a case, and the symptoms at admission) and in the calculation of the age in months. The category Patient for study required the LLM to combine two facts about a patient: The age, which had to be between 2 and 5 years (inclusive), and whether or not the patient was admitted due to a probable or proven infectious disease. Some LLMs showed a lower performance in this combined category than could be expected based on the results from the sub-categories (age and infectious disease).

## Discussion

The presented data suggests that LLMs are capable of extracting structured information from unstructured clinical reports. The results show several tendencies.

Regarding overall performance, the commercial GP4o from the OpenAI family had a higher evaluation score than all other LLMs. The smaller OpenAI model, GPT4o-mini, had a performance that was similar to the bigger open-source LLMs that can be run locally. Among the local LLMs, there was a tendency that models with a higher number of parameters achieved higher scores. All models with more than 8 billion parameters had a score of more than 80. There is one exception: The LLM Llama3-Med42 with 70 billion parameters, which is a fine tune of Llama 3, had a score of 74.5, which is in the range of the models with eight billion parameters. A detailed analysis of these results showed that Llama3-Med42 sometimes failed to deliver the replies in a valid JSON format. This could be an instance of the so-called “catastrophic forgetting”, i.e. the losing of prior information (in this case: to produce valid machine-readable JSON data) upon learning new information (here: medical knowledge) [13].

All categories that only involved data extraction and simple classification (infectious disease yes/no) were processed perfectly or near-perfectly by the best OpenAI model as well as by some of the local LLMs. Solving complex calculations (the age in months) did not yield satisfactory results for any LLM. In two tasks, the LLMs were asked to list the presenting symptoms and the involved sub-specialties for a given patient. Here, the results were in the lower range for all LLMs. It is noteworthy that the inter-rater reliability for humans in similar tasks has been shown to be far from perfect [14].

These results align with the current literature. In a recent study, LLMs from the “LLAMA 2” family of open-source models were able to identify abdominal pain, shortness of breath, confusion, liver cirrhosis, and ascites from medical histories with accuracies above 90%, even if the symptoms were not explicitly mentioned in the text [7]. These results are similar to those presented here: It seems that the LLMs are well suited to answer yes/no questions on medical texts, even if some degree of medical knowledge is required to do so. A study evaluated the performance of the closed-source OpenAI LLM “GPT-4” on the analysis of histopathology reports [15]. The results of this study were very also very promising. When analyzing radiology reports, GPT-4 outperformed the state-of-the art model in another study [16].

There are several limitation to this study. Firstly, the LLMs were evaluated on fictional letters. Care has been taken to create a realistic sample of fictional patients, and to include the writing style of different pediatricians. Still, the performance on real-world data might differ. Future studies should validate these findings on authentic, anonymized clinical data. Secondly, the study provided a comparatively small number of example letters. And finally, it should be noted that the selection of the LLMs used in this study was subjective. The aim was to present the performance of both commercial, closed-source LLMs, and of recent open-source LLMs of varying sizes and types. Therefore, most LLMs that are available were not tested and their results might vary.

Nonetheless, the results presented here hint on a general usability of both closed-source and open-source LLMs for extracting structured data from clinical reports, even if these letter were given in German instead of the nominal standard language of the LLMs that was English. In principle, all models shared the same characteristics. The performance of the LLMs was highest in the extraction of a single, unambiguous data point (i.e. the body weight) and in straightforward assessments that required some medical knowledge from the LLM (such as determining whether or not a genetic disorder or a infectious disease was present). This demonstrates their potential for automating routine clinical documentation tasks, reducing manual workload for healthcare professionals. Complex numerical calculations resulted in a higher number of errors, likely reflecting limitations in the models’ numerical reasoning capabilities, which are not their primary design focus. This suggests a need for additional fine-tuning or hybrid approaches combining LLMs with traditional algorithms for such tasks.

What are the practical consequences of these results? In general, LLMs can extract structured data from clinical reports. Depending on the specific task, some of the error rates are too high for a use in real-world patient care. But, even today, LLMs can be used as pre-screening tools for clinical trials, automatically flagging patients based on predefined criteria, or as assistants for generating structured summaries of clinical notes, thereby enhancing the efficiency of electronic health record management. LLMs are a young technology, and future LLMs can be assumed to perform better. Additionally, exploring hybrid systems that combine LLMs with domain-specific algorithms could enhance their reliability and broaden their applicability in clinical and research settings.

## Supporting information

S1 Fig Comparison between the age distribution in the study population and the real-world age distribution of all patients admitted to pediatric ward No. 4 at Dr. von Hauner Children’s Hospital, Munich, Germany, between April 1, 2024, and March 31, 2025. (TIF)

S1 File All source data files used for the study, including the fictional medical reports and the classifications thereof. (TGZ)
