# Supplementary figures and images for: Using large language models to extract information from pediatric clinical reports
Source: PLOS Digit Health. 2025 Jul 23;4(7):e0000919. doi: 10.1371/journal.pdig.0000919 (PMC12286318; doi:10.1371/journal.pdig.0000919)

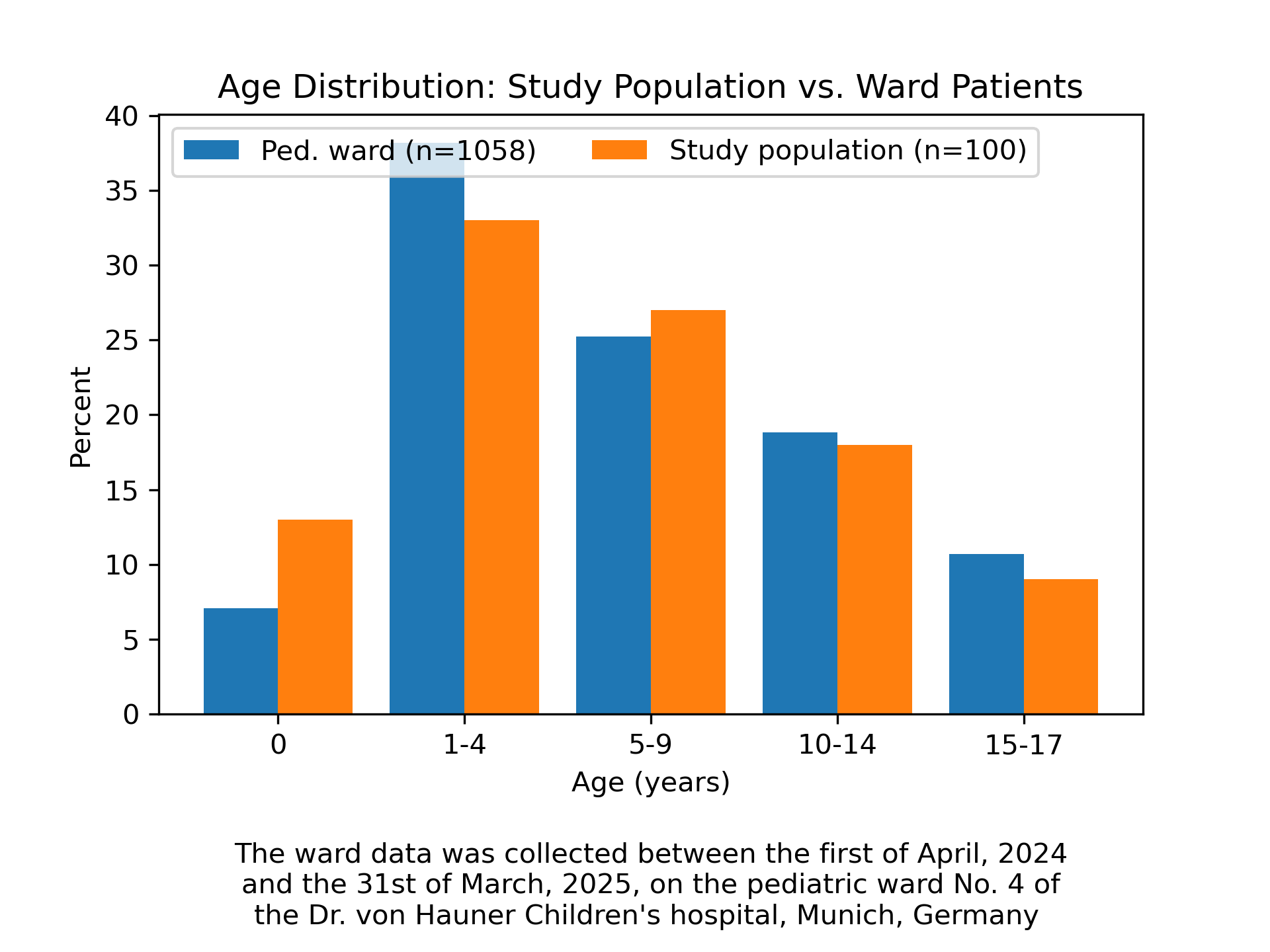

Supplement: S1 Fig — (TIF) [file pdig.0000919.s001.tif]
